# Crystal structure and anti­mycobacterial evaluation of 2-(cyclo­hexyl­meth­yl)-7-nitro-5-(tri­fluoro­meth­yl)benzo[*d*]iso­thia­zol-3(2*H*)-one^1^

**DOI:** 10.1107/S2056989023010137

**Published:** 2023-11-30

**Authors:** Adrian Richter, Richard Goddard, Peter Imming, Rüdiger W. Seidel

**Affiliations:** a Institut für Pharmazie, Martin-Luther-Universität Halle-Wittenberg, Wolfgang-Langenbeck-Str. 4, 06120 Halle (Saale), Germany; b Max-Planck-Institut für Kohlenforschung, Kaiser-Wilhelm-Platz 1, 45470 Mülheim an der Ruhr, Germany; Katholieke Universiteit Leuven, Belgium

**Keywords:** benziso­thia­zolinone, benzo­thia­zinone, mycobacteria, hydrogen bonding, crystal structure

## Abstract

The title compound, C_15_H_15_F_3_N_2_O_3_S, crystallizes in the monoclinic system, space group *I*2/*a*, with *Z* = 8. As expected, the nine-membered heterobicyclic system is virtually planar and the cyclo­hexyl group adopts a chair conformation. There is structural evidence for intra­molecular N—S⋯O chalcogen bonding between the benziso­thia­zolinone S atom and one O atom of the nitro group, approximately aligned along the extension of the covalent N—S bond [N—S⋯O = 162.7 (1)°]. In the crystal, the mol­ecules form centrosymmetric dimers through C—H⋯O weak hydrogen bonding between a C—H group of the electron-deficient benzene ring and the benzo­thia­zolinone carbonyl O atom with an *R* ^2^_2_(10) motif. In contrast to the previously described *N*-acyl 7-nitro-5-(tri­fluoro­meth­yl)benzo[*d*]iso­thia­zol-3(2*H*)-ones, the title *N*-cyclo­hexyl­methyl analogue does not inhibit growth of *Mycobacterium aurum* and *Mycobacterium smegmatis in vitro*.

## Abstract 2

The crystal structure of the title compound features centrosymmetric dimers formed through C—H⋯O weak hydrogen bonds between a C—H group of the electron-deficient benzene ring and the benzo­thia­zolinone carbonyl O atom with an 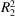 (10) motif.

## 1. Chemical context

**Figure 1:**
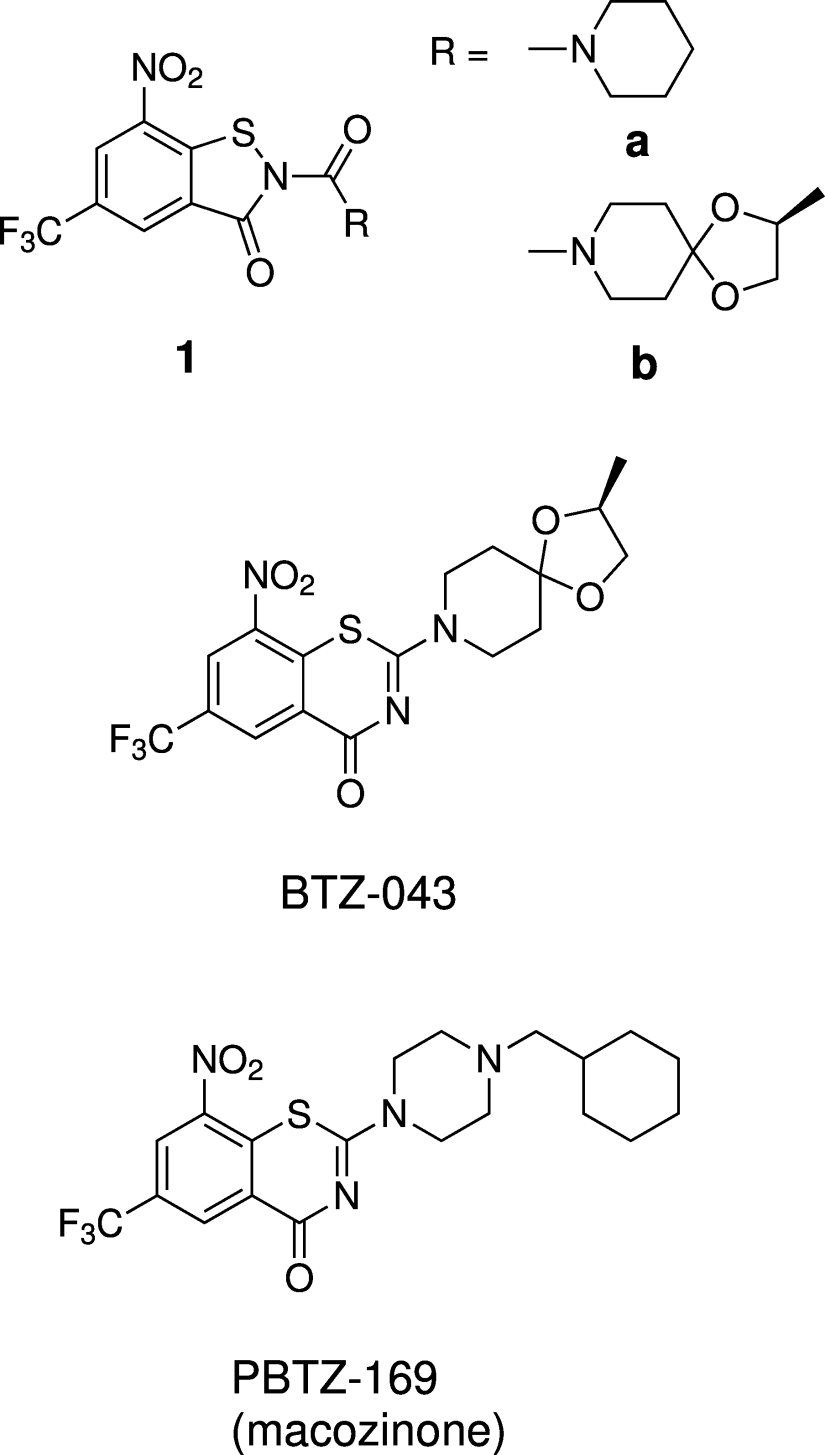
Chemical diagrams of previously reported BITs **1a** and **1b** exhibiting *in vitro* anti­mycobacterial activity (Richter *et al.*, 2022 ▸) and anti­tubercular BTZs that have advanced to clinical studies (Makarov & Mikušová, 2020 ▸).

**Figure 2:**
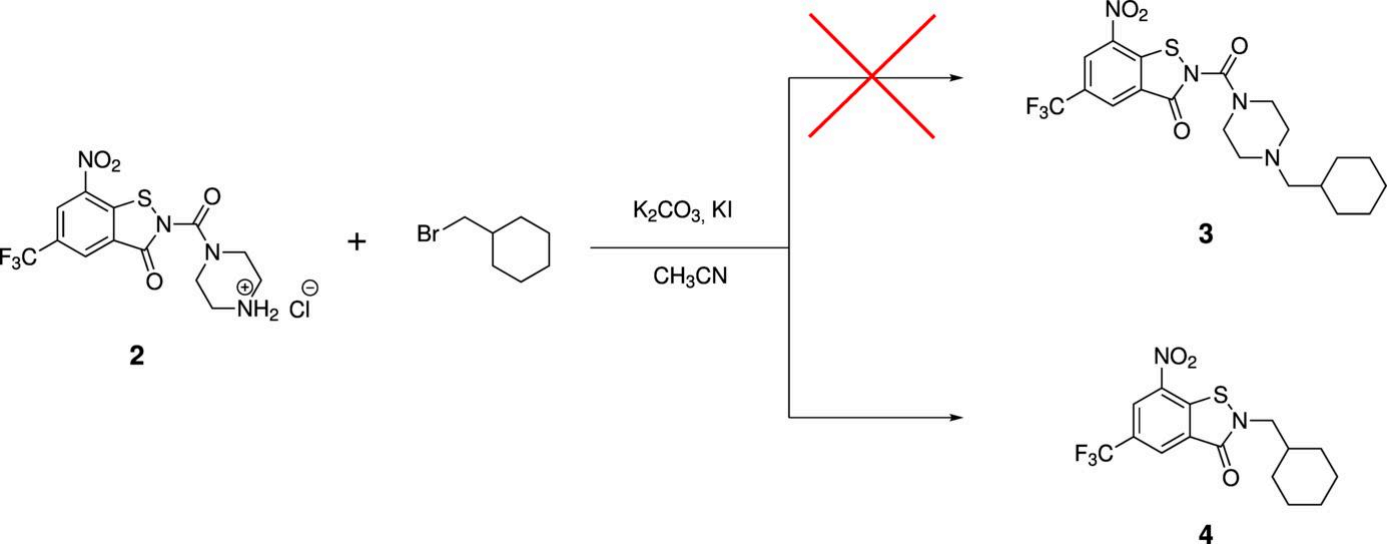
Unintentional formation of **4**, the title compound, from the 2-(piperazine-1-carbon­yl)-BIT **2** and cyclo­hexyl­methyl bromide.

Benziso­thia­zolinones (BITs) are known to exhibit broad-spectrum anti­microbial effects (Gopinath *et al.*, 2017 ▸). The unsubstituted BIT and other iso­thia­zolinones are widely used as biocides (Silva *et al.*, 2020 ▸). In the course of our quest for new anti­mycobacterial agents, we recently reported the *N*-acyl BITs **1a** and **1b** (Fig. 1 ▸). They displayed *in vitro* activity against mycobacteria including *Mycobacterium tuberculosis* (Richter *et al.*, 2022 ▸), the major etiological agent of tuberculosis. Together with the corresponding *S*-oxides, they were originally discovered by chance in an attempt to oxidize benzo­thia­zinones at the S atom (BTZs; Eckhardt *et al.*, 2020 ▸). BTZs, in particular 8-NO_2_-BTZs, are a promising class of anti­tuberculosis drug candidates (Seidel *et al.*, 2023 ▸), two of which have progressed to clinical studies, *viz.* BTZ-043 and PBTZ-169 (Fig. 1 ▸; Makarov & Mikušová, 2020 ▸). The pyridine-1-carbonyl *spiro* ketal side chain appended to the N atom in 2-position of the BIT scaffold in **1b** is inspired by that of BTZ-043. In an attempt to synthesize the analogous BIT **3** bearing the PBTZ-169-inspired piperazin-1-carbonyl side chain from the precursor **2** and cyclo­hexyl­methyl bromide, we unintentionally obtained **4**, the title compound (Fig. 2 ▸). 
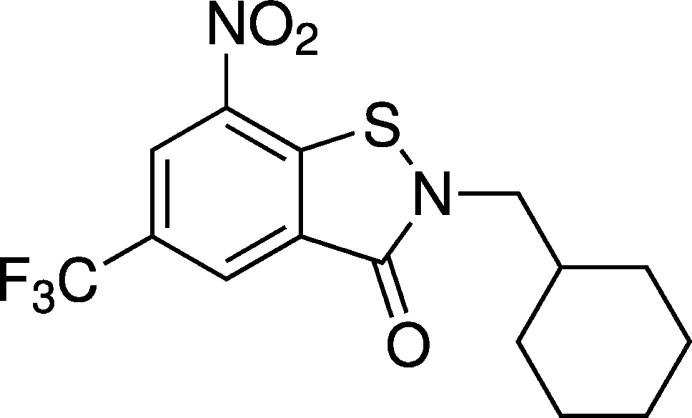


## 2. Structural commentary

**Figure 3:**
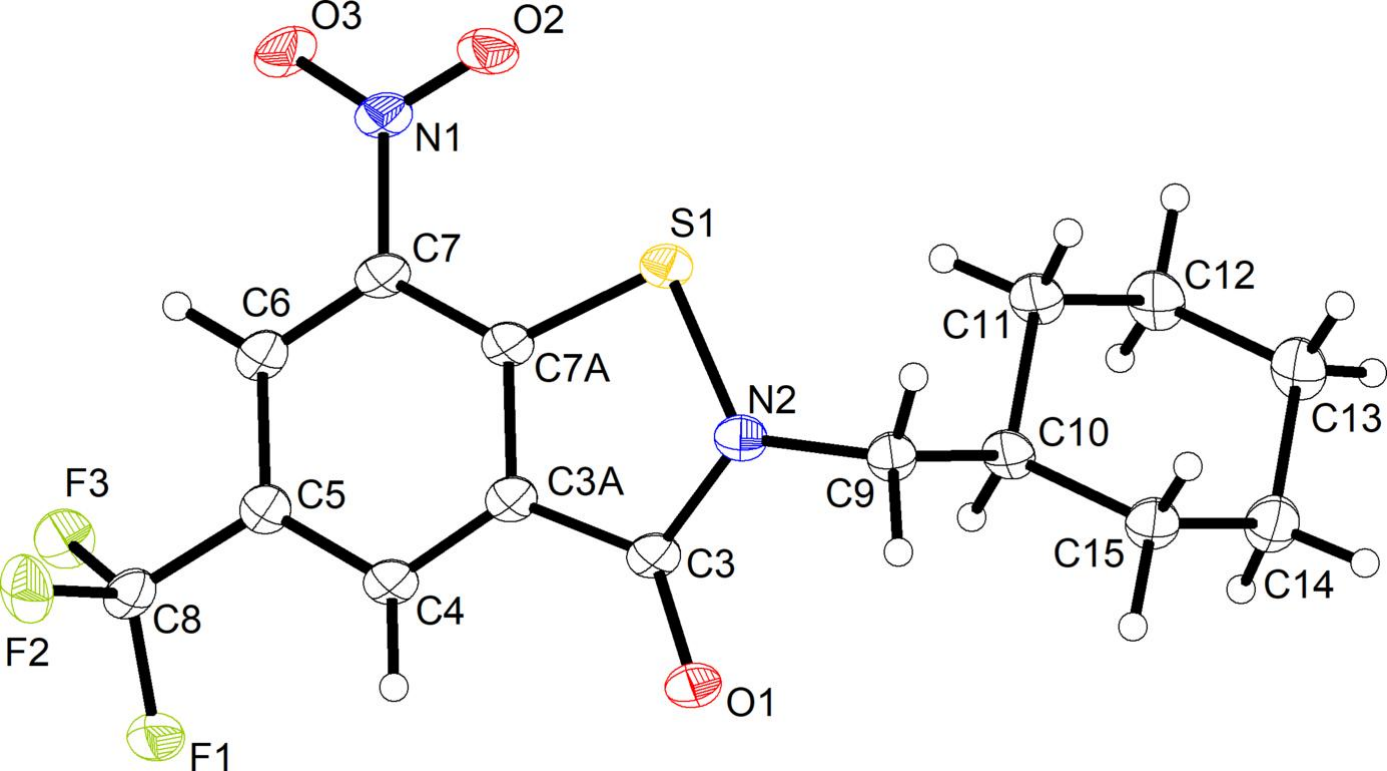
Mol­ecular structure of **4**. Displacement ellipsoids are drawn at the 50% probability level. H atoms are represented by small spheres of arbitrary radius.

Fig. 3 ▸ shows the mol­ecular structure of **4** in the crystal, and Table 1 ▸ lists selected bond lengths and angles. The nine-membered heterobicyclic system is virtually planar with a r.m.s. deviation of 0.0294 Å. The C—C—C bond angles within the benzene ring alternate in magnitude by *ca* ±2°, with the larger angles being associated with the C atoms bonded to electron-withdrawing groups, *viz.* C(=O)N, NO_2_ and CF_3_. The somewhat long C3—O1 distance of 1.226 (3) Å is consistent with the relatively low wavenumber of the carbonyl band at 1630 cm^−1^ in the IR spectrum (see supporting information), which is typical of amides. The dihedral angle between the BIT mean plane and the plane defined by the three atoms of the nitro group is 11.4 (3)°. The intra­molecular S1⋯O2 distance of 2.603 (2) Å and the N2—S1⋯O2 angle of 162.74 (8)° suggest the existence of an intra­molecular chalcogen bond on the extension of the covalent N—S bond (Scilabra *et al.*, 2019 ▸; Vogel *et al.*, 2019 ▸; Pizzi *et al.*, 2023 ▸). The orientation of the BIT moiety and the cyclo­hexyl­methyl group to one another renders the mol­ecule axially chiral, although the centrosymmetric crystal structure contains both enanti­omeric conformers. The cyclo­hexyl group adopts a low-energy chair conformation with the C—C—C bond angles being close to the ideal tetra­hedral angle (Table 1 ▸).

## 3. Supra­molecular features

**Figure 4:**
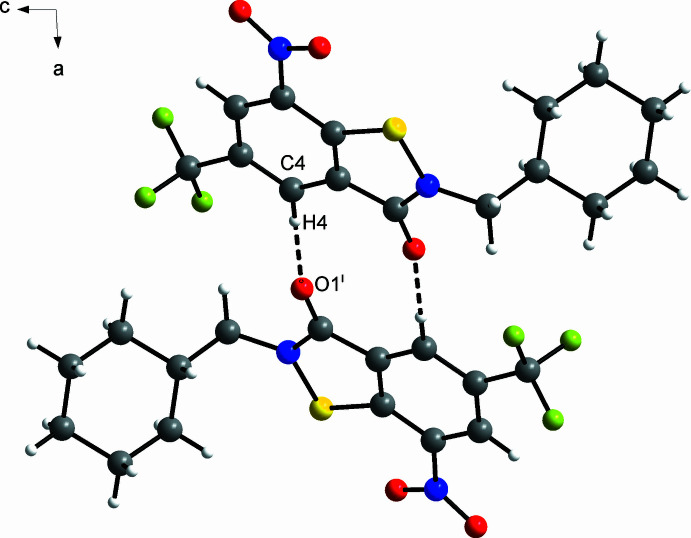
Centrosymmetric C—H⋯O hydrogen-bonded dimer of **4** in the crystal, viewed along the *b-*axis direction towards the origin. Dashed lines represent hydrogen bonds. Colour scheme: C, grey; H, white; F, green; N, blue; O, red; S, yellow. Symmetry code: (i) −*x* + 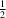 , −*y* − 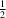 , −*z* + 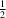 .

**Figure 5:**
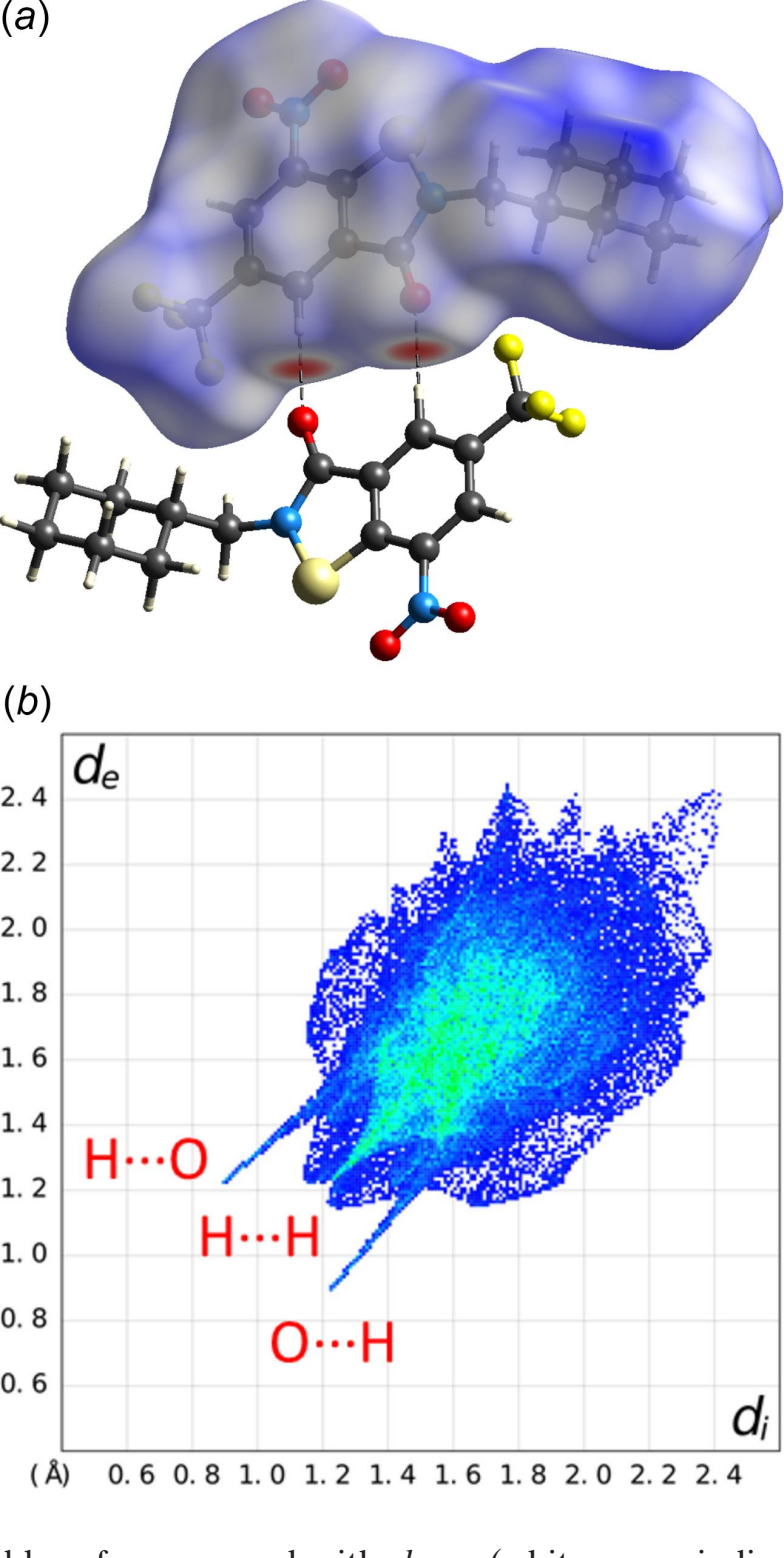
(*a*) Hirshfeld surface mapped with *d*_norm_ (white areas indicate van der Waals contacts, red areas shorter-than and blue areas longer-than van der Waals contacts) and (*b*) the corresponding two-dimensional fingerprint plot; *d*_i_ and *d*_e_ are the respective inter­ior and exterior distances of the nearest atom to the Hirshfeld surface over the range 0.4–2.6 Å (blue, few points; green, moderate fraction; red, many points). The figure was generated with *CrystalExplorer* (Spackman *et al.*, 2021 ▸).

**Figure 6:**
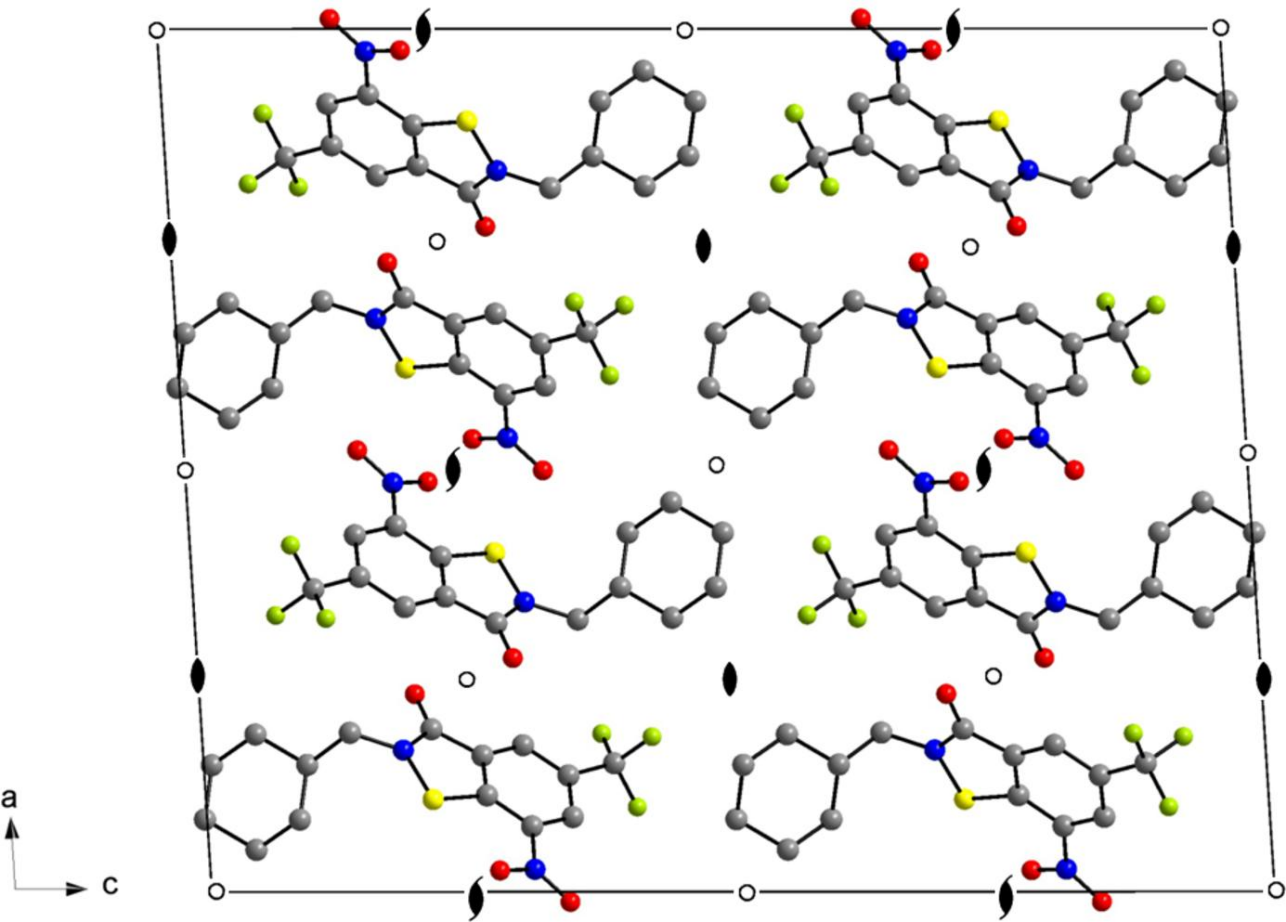
Packing diagram of **4**, viewed along the *b*-axis direction. H atoms have been omitted for clarity. Colour scheme: C, grey; F, green; N, blue; O, red; S, yellow.

The most prominent supra­molecular feature of the crystal structure of **4** is weak inter­molecular C—H⋯O hydrogen bonding. As shown in Fig. 4 ▸, the mol­ecules form centrosymmetric dimers through C—H⋯O hydrogen bonds between the C4—H4 moiety of the benzene ring and the carbonyl O atom of an adjacent symmetry-related mol­ecule. The C4—H4 group is likely activated for weak hydrogen bonding through the electron-withdrawing effect exerted by the C(=O)N and CF_3_ groups in *ortho* positions and the NO_2_ group in the *para* position. The graph-set descriptor for the hydrogen-bond motif is 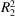 (10) (Bernstein *et al.*, 1995 ▸). Table 2 ▸ lists the corresponding geometric parameters, which are typical of weak hydrogen bonds (Thakuria *et al.*, 2017 ▸). The dominance of the short O⋯H contacts is also revealed by a Hirshfeld surface analysis (Spackman & Jayatilaka, 2009 ▸), as shown in Fig. 5 ▸. In addition, H⋯H contacts, mostly resulting from close packing of the cyclo­hexyl groups, as shown in Fig. 6 ▸, are evident. The packing index (Kitaigorodskii, 1973 ▸) of the crystal structure, as calculated with *PLATON* (Spek, 2020 ▸), is 71.6%. Notably, the crystal structure also features a short inter­molecular O⋯N contact (2.87 Å) between adjacent mol­ecules related by 2_1_ screw symmetry. Inter­molecular F⋯F contacts between CF_3_ groups are not encountered.

## 4. Database survey

As of November 2023, a search of the Cambridge Structural Database (CSD; Groom *et al.*, 2016 ▸) reveals more than 50 crystal structures containing a BIT scaffold. Specifically, two 7-NO_2_-5-CF_3_-BITs with 2-(piperidine-1-carbon­yl) side chains and their benziso­thia­zol-3-ol constitutional isomers (Richter *et al.*, 2022 ▸) as well as the corresponding BIT 1-oxides (Eckhardt *et al.*, 2020 ▸) have previously been structurally characterized by us. Of note, the centrosymmetric C—H⋯O weak hydrogen-bond-dimer motif encountered in the crystal structure of **4** is not found in the BIT structures contained in the CSD. For a data-mining survey of the CSD for a statistical assessment of the chalcogen bond ability of the sulfur atom in BITs and related compounds, we direct the inter­ested reader to the recent publication by Pizzi *et al.* (2023 ▸).

## 5. Anti­mycobacterial evaluation

Compound **4** was subjected to *in vitro* testing against *Mycobacterium aurum* and *Mycobacterium smegmatis* using the broth microdilution method, as described previously (Richter *et al.*, 2022 ▸). The generally considered non-pathogenic mycobacterial species *M. aurum* (Gupta *et al.*, 2009 ▸; Namouchi *et al.*, 2017 ▸; Phelan *et al.*, 2015 ▸) and *M. smegmatis* (Sundarsingh *et al.*, 2020 ▸) have been used as surrogate bacteria in early-stage anti­tuberculosis drug discovery. Using Middlebrook 7H9 liquid growth medium supplemented with 10% ADS [5% (*m*/*v*) bovine serum albumin fraction V, 0.81% (*m*/*v*) sodium chloride and 2% (*m*/*v*) dextrose in purified water] and 0.05% polysorbate 80, we found no *in vitro* activity of **4** against *M. aurum* DSM 43999 and *M. smegmatis* mc^2^ 155 up to 100 μ*M*. The findings essentially confirm that, similar to anti­tubercular BTZs (Seidel *et al.*, 2023 ▸), the nature of the side chain appended to the N atom in position 2 of the BIT scaffold has a crucial bearing on the anti­mycobacterial activity (Richter *et al.*, 2022 ▸).

## 6. Synthesis and crystallization

*General:* Chemicals were of reagent-grade quality and used as received. 7-Nitro-5-(tri­fluoro­meth­yl)benzo[*d*]iso­thia­zol-3(2*H*)-one was prepared as described previously (Richter *et al.*, 2022 ▸). Solvents were distilled prior to use and stored over 4 Å mol­ecular sieves. Flash chromatography was performed on an Inter­chim puriFlash 430 instrument. NMR spectra were recorded on an Agilent Technologies VNMRS 400 MHz or a Varian INOVA 500 MHz NMR spectrometer. ^1^H and ^13^C chemical shifts are reported relative to the residual solvent signal of CDCl_3_ (δ_H_ = 7.26 ppm; δ_C_ = 77.10 ppm) or CD_3_OD (δ_H_ = 4.78 ppm). The ^19^F chemical shifts are reported relative to the signal of CFCl_3_ (δ_F_ = 0 ppm) as an external standard. HPLC analysis was conducted with a Shimadzu instrument with a CBM-40 control unit, two LC-40D pumps and an SPD-M40 PDA UV detector, using an Agilent Poroshell 120, EC-C18, 3.0 × 50 mm, 2.7 µm column at a flow rate of 1.2 mL min^−1^, eluting with water/aceto­nitrile. APCI mass spectrometry was carried out on an Advion Expression compact mass spectrometer using the direct analysis probe method. The ESI mass spectrum was measured on a Thermo Scientific Q Exactive^TM^ Plus Orbitrap mass spectrometer and the EI mass spectrum on a Finnigan MAT 95 mass spectrometer. The IR spectrum was recorded on a Bruker Tensor II Platinum ATR spectrometer at a resolution of 4 cm^−1^, accumulating 16 scans.

*Synthesis of 2-(4-Boc-piperazine-1-carbon­yl)-7-nitro-5-(tri­fluoro­meth­yl)benzo[d]iso­thia­zol-3(2H)-one:* 7-Nitro-5-(tri­fluoro­meth­yl)benzo[*d*]iso­thia­zol-3(2*H*)-one (600 mg, 2.27 mmol) and 4-Boc-1-piperazinecarbonyl chloride were dissolved in 35 mL of di­chloro­methane. Pyridine (1.83 mL, 22.7 mmol, 10.0 eq.) was added and the reaction mixture was stirred for 24 h at room temperature. After removal of the solvent *in vacuo*, the product was isolated as a yellow solid by flash chromatography on silica gel (ethyl acetate/heptane gradient), eluting after the *O*-acyl­ated constitutional isomer major product (*vide infra*). Yield: 152 mg (0.32 mmol, 14%). ^1^H NMR (500 MHz, CDCl_3_): δ 8.79 (*m*, *J* = 0.9 Hz, 1H), 8.57 (*m*, *J* = 1.0 Hz, 1H), 3.61 (*s*, 8H), 1.49 (*s*, 9H) ppm. ^13^C NMR (101 MHz, CDCl_3_): δ 161.1, 154.6, 150.0, 142.3, 140.5, 130.5 (*q*, ^3^ *J* = 3.7 Hz), 129.9 (*q*, ^2^ *J* = 35 Hz), 128.3, 125.8 (*q*, ^3^ *J* = 4 Hz), 122.5 (*q*, ^1^ *J* = 273 Hz), 80.7, 46.8, 43.5, 28.5. ^19^F NMR (376 MHz, CDCl_3_): δ −62.18 ppm. MS(APCI^+^): *m*/*z* calculated for C_18_H_20_F_3_N_4_O_6_S^+^: 477.1; found: 476.9 [*M*+H^+^]. The con­sti­tutional isomer *7-nitro-5-(tri­fluoro­meth­yl)benzo[d]iso­thia­zol-3-yl 4-Boc-piperazine-1-carboxyl­ate* was isolated as major product in 76% yield (823 mg, 1.72 mmol). ^1^H NMR (500 MHz, CDCl_3_): δ 8.73 (*m*, 1H), 8.42 (*m*, 1H), 3.76 (*m*, 2H), 3.67–3.52 (*m*, 6H), 1.50 (*s*, 9H) ppm. ^13^C NMR (126 MHz, CDCl_3_): δ 156.9, 154.6, 151.0, 149.5, 141.4, 129.9, 129.4 (*q*, ^2^ *J* = 35 Hz), 126.9 (*q*, ^3^ *J* = 4 Hz), 122.8 (*q*, ^1^ *J* = 273 Hz), 121.9 (*q*, ^3^ *J* = 3 Hz), 80.8, 45.2, 44.5, 43.4, 28.5 ppm. ^19^F NMR (470 MHz, CDCl_3_): δ −61.59 ppm. MS(APCI^+^): *m*/*z* calculated for C_18_H_20_F_3_N_4_O_6_S^+^: 477.1; found: 476.9 [*M*+H^+^].

*Synthesis of 4-(7-nitro-3-oxo-5-(tri­fluoro­meth­yl)-2,3-di­hydro­benzo[d]iso­thia­zole-2-carbon­yl)piperazin-1-ium chlor­ide (**2**):* 2-(4-Boc-piperazine-1-carbon­yl)-7-nitro-5-(tri­fluorometh­yl)benzo[*d*]iso­thia­zol-3(2*H*)-one was dissolved in 1 mL of 4 *M* HCl in 1,4-dioxane. After stirring overnight at room temperature, compound **2** was collected by filtration to yield 86 mg (0.21 mmol, 65%). ^1^H NMR (400 MHz, CD_3_OD): δ 8.93 (*m*, 1H), 8.62 (*m*, 1H), 3.90–3.85 (*m*, 4H), 3.46–3.40 (*m*, 4H). MS(APCI^+^): *m*/*z* calculated for C_13_H_12_F_3_N_4_O_4_S^+^: 377.1; found: 376.9 [*M*+H^+^].

*Synthesis of 2-(cyclo­hexyl­meth­yl)-7-nitro-5-(tri­fluoro­meth­yl)benzo[d]iso­thia­zol-3(2H)-one (**4**):* Compound **2** (50 mg, 0.12 mmol), cyclo­hexyl­methyl bromide (18 µL, 0.13 mmol, 1.1 eq.), potassium iodide (∼1 mg) and potassium carbonate (20 mg, 0.14 mmol, 1.2 eq.) were suspended in 5 mL of aceto­nitrile. The reaction mixture was stirred at room temperature and the progress of the reaction was monitored by TLC. After the starting material had been consumed, the solvent was removed *in vacuo*. The residue was subjected to flash chromatography on silica gel (chloro­form/heptane gradient) to yield **4** as a yellow powder (14 mg, 0.04 mmol, 33%, HPLC purity >97%). ^1^H NMR (400 MHz, CDCl_3_): δ 8.70 (*m*, 1H), 8.48 (*m*, 1H), 4.39 (*d*, *J* = 6.4 Hz, 2H), 2.00–1.88 (*m*, 3H), 1.84–1.71 (*m*, 3H), 1.38–1.24 (*m*, 3H), 1.18–1.08 (*m*, 2H). ^13^C NMR (101 MHz, CDCl_3_) δ 163.3, 149.4, 141.2, 129.1, 128.6 (*q*, ^2^ *J*_C,F_ = 35 Hz), 126.7 (*q*, ^3^ *J*_C,F_ = 4 Hz), 124.4 (*q*, ^1^ *J*_C,F_ = 273 Hz), 122.0 (*q*, ^3^ *J*_C,F_ = 4 Hz), 75.3, 37.5, 29.8, 26.5, 25.8. HRMS(ESI^+^): *m*/*z* calculated for C_15_H_16_F_3_N_2_O_3_S^+^: 361.08282; found: 361.08264 [*M*+H]^+^. MS(EI^+^): *m*/*z* calculated for C_15_H_15_F_3_N_2_O_3_S^+^: 360; found: 360 [*M* ^+^]. IR(ATR): ν^~^ 1630 (C=O). Yellow needles of **4** suitable for single-crystal X-ray diffraction analysis were obtained when a chloro­form/heptane solution of the compound slowly evaporated to dryness at room temperature.

## 7. Refinement

Crystal data, data collection and structure refinement details are given in Table 3 ▸. H atoms were placed in geometrically calculated positions and refined using the appropriate riding model, with C_aromatic_—H = 0.95 Å, C_methyl­ene_—H = 0.99 Å, C_methine_—H = 1.00 Å and *U*_iso_(H) = 1.2*U*_eq_(C).

## Supplementary Material

Crystal structure: contains datablock(s) global, I. DOI: 10.1107/S2056989023010137/vm2292sup1.cif

Structure factors: contains datablock(s) I. DOI: 10.1107/S2056989023010137/vm2292Isup2.hkl

Supporting information file. DOI: 10.1107/S2056989023010137/vm2292Isup3.cdx

NMR, MS, IR and HPLC data associated with the article. DOI: 10.1107/S2056989023010137/vm2292sup4.pdf

Supporting information file. DOI: 10.1107/S2056989023010137/vm2292Isup5.cml

CCDC reference: 2309724

Additional supporting information:  crystallographic information; 3D view; checkCIF report

## Figures and Tables

**Table 1:** Selected geometric parameters (Å, °)

C3—O1	1.226 (3)	C5—C6	1.395 (3)
C3—N2	1.366 (3)	C5—C8	1.488 (3)
C3—C3*A*	1.463 (3)	C6—C7	1.374 (3)
C3*A*—C4	1.384 (3)	C7—C7*A*	1.391 (3)
C3*A*—C7*A*	1.402 (3)	C7—N1	1.448 (3)
C4—C5	1.388 (3)	C7*A*—S1	1.706 (2)
			
N2—C3—C3*A*	108.44 (19)	C12—C11—C10	111.2 (2)
C4—C3*A*—C7*A*	121.2 (2)	C13—C12—C11	111.3 (2)
C4—C3*A*—C3	126.9 (2)	C12—C13—C14	110.9 (2)
C7*A*—C3*A*—C3	112.0 (2)	C15—C14—C13	111.2 (2)
C6—C7—C7*A*	121.8 (2)	C14—C15—C10	111.6 (2)
C7—C7*A*—C3*A*	118.1 (2)	C3—N2—S1	116.46 (16)
C3*A*—C7*A*—S1	112.84 (18)	C9—N2—S1	121.45 (16)
C15—C10—C11	110.53 (19)	C7*A*—S1—N2	90.24 (10)

**Table 2:** Hydrogen-bond geometry (Å, °)

*D*—H⋯*A*	*D*—H	H⋯*A*	*D*⋯*A*	*D*—H⋯*A*
C4—H4⋯O1^i^	0.95	2.25	3.193 (3)	175

Symmetry code: (i) 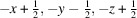 .

**Table 3:** Experimental details

Crystal data	
Chemical formula	C_15_H_15_F_3_N_2_O_3_S
*M*_r_	360.35
Crystal system, space group	Monoclinic, *I*2/*a*
Temperature (K)	100
*a*, *b*, *c* (Å)	21.9709 (15), 5.1271 (4), 27.022 (2)
β (°)	93.633 (4)
*V* (Å^3^)	3037.8 (4)
*Z*	8
Radiation type	Mo *K*α
μ (mm^−1^)	0.27
Crystal size (mm)	0.20 × 0.06 × 0.06

Data collection	
Diffractometer	Bruker Kappa Mach3 APEXII
Absorption correction	Gaussian (*SADABS*; Krause *et al.*, 2015 ▸)
*T*_min_, *T*_max_	0.967, 0.992
No. of measured, independent and observed [*I* > 2σ(*I*)] reflections	61976, 2836, 2243
*R*_int_	0.100
(sin θ/λ)_max_ (Å^−1^)	0.606

Refinement	
*R*[*F* ^2^ > 2σ(*F* ^2^)], *wR*(*F* ^2^), *S*	0.042, 0.116, 1.04
No. of reflections	2836
No. of parameters	217
H-atom treatment	H-atom parameters constrained
Δρ_max_, Δρ_min_ (e Å^−3^)	0.34, −0.33

Computer programs: *APEX3* (Bruker, 2017 ▸), *SAINT* (Bruker, 2004 ▸), *SHELXT2014/5* (Sheldrick, 2015*a* ▸), *SHELXL2019/3* (Sheldrick, 2015*b* ▸), *DIAMOND* (Brandenburg, 2018 ▸), *enCIFer* (Allen *et al.*, 2004 ▸) and *publCIF* (Westrip, 2010 ▸).

## supplementary crystallographic information

### Crystal data

**Table:**

C_15_H_15_F_3_N_2_O_3_S	*F*(000) = 1488
*M**_r_* = 360.35	*D*_x_ = 1.576 Mg m^−^^3^
Monoclinic, *I*2/*a*	Mo *K*α radiation, λ = 0.71073 Å
*a* = 21.9709 (15) Å	Cell parameters from 9947 reflections
*b* = 5.1271 (4) Å	θ = 2.5–28.4°
*c* = 27.022 (2) Å	µ = 0.27 mm^−^^1^
β = 93.633 (4)°	*T* = 100 K
*V* = 3037.8 (4) Å^3^	Needle, yellow
*Z* = 8	0.20 × 0.06 × 0.06 mm

### Data collection

**Table:**

Bruker Kappa Mach3 APEXII diffractometer	2836 independent reflections
Radiation source: microfocus X-ray source	2243 reflections with *I* > 2σ(*I*)
Incoatec Helios mirrors monochromator	*R*_int_ = 0.100
Detector resolution: 66.67 pixels mm^-1^	θ_max_ = 25.5°, θ_min_ = 1.5°
φ and ω scans	*h* = −26→26
Absorption correction: gaussian (SADABS; Krause *et al.*, 2015)	*k* = −6→6
*T*_min_ = 0.967, *T*_max_ = 0.992	*l* = −32→32
61976 measured reflections	

### Refinement

**Table:**

Refinement on *F*^2^	Primary atom site location: dual
Least-squares matrix: full	Secondary atom site location: difference Fourier map
*R*[*F*^2^ > 2σ(*F*^2^)] = 0.042	Hydrogen site location: inferred from neighbouring sites
*wR*(*F*^2^) = 0.116	H-atom parameters constrained
*S* = 1.04	*w* = 1/[σ^2^(*F*_o_^2^) + (0.0554*P*)^2^ + 5.9357*P*] where *P* = (*F*_o_^2^ + 2*F*_c_^2^)/3
2836 reflections	(Δ/σ)_max_ < 0.001
217 parameters	Δρ_max_ = 0.34 e Å^−^^3^
0 restraints	Δρ_min_ = −0.33 e Å^−^^3^

### Special details

**Table:**

Geometry. All esds (except the esd in the dihedral angle between two l.s. planes) are estimated using the full covariance matrix. The cell esds are taken into account individually in the estimation of esds in distances, angles and torsion angles; correlations between esds in cell parameters are only used when they are defined by crystal symmetry. An approximate (isotropic) treatment of cell esds is used for estimating esds involving l.s. planes.

### Fractional atomic coordinates and isotropic or equivalent isotropic displacement parameters (Å^2^)

**Table:**

	*x*	*y*	*z*	*U*_iso_*/*U*_eq_	
C3	0.18859 (9)	0.1371 (5)	0.21632 (9)	0.0197 (5)	
C3A	0.15790 (10)	0.1247 (5)	0.26275 (8)	0.0183 (5)	
C4	0.16991 (10)	−0.0481 (5)	0.30149 (9)	0.0197 (5)	
H4	0.201655	−0.173215	0.300101	0.024*	
C5	0.13485 (10)	−0.0359 (5)	0.34241 (9)	0.0198 (5)	
C6	0.08669 (10)	0.1406 (5)	0.34402 (9)	0.0202 (5)	
H6	0.061716	0.142709	0.371460	0.024*	
C7	0.07599 (10)	0.3113 (5)	0.30523 (9)	0.0196 (5)	
C7A	0.11180 (9)	0.3127 (5)	0.26458 (8)	0.0186 (5)	
C8	0.14904 (10)	−0.2056 (5)	0.38629 (9)	0.0217 (5)	
C9	0.18439 (10)	0.4007 (5)	0.13941 (8)	0.0207 (5)	
H9A	0.227784	0.350627	0.138129	0.025*	
H9B	0.181497	0.591480	0.134170	0.025*	
C10	0.14732 (10)	0.2631 (5)	0.09739 (8)	0.0209 (5)	
H10	0.141760	0.077299	0.107440	0.025*	
C11	0.08388 (10)	0.3832 (5)	0.08715 (9)	0.0234 (5)	
H11A	0.060785	0.370255	0.117343	0.028*	
H11B	0.088125	0.570227	0.078966	0.028*	
C12	0.04881 (10)	0.2450 (6)	0.04447 (9)	0.0278 (6)	
H12A	0.041006	0.062157	0.053996	0.033*	
H12B	0.008923	0.331937	0.037643	0.033*	
C13	0.08402 (11)	0.2483 (6)	−0.00212 (9)	0.0283 (6)	
H13A	0.088292	0.430440	−0.013581	0.034*	
H13B	0.061107	0.149498	−0.028697	0.034*	
C14	0.14704 (11)	0.1280 (6)	0.00764 (9)	0.0278 (6)	
H14A	0.142728	−0.059395	0.015561	0.033*	
H14B	0.169975	0.141924	−0.022619	0.033*	
C15	0.18246 (10)	0.2642 (5)	0.05047 (9)	0.0245 (6)	
H15A	0.190777	0.446672	0.040981	0.029*	
H15B	0.222109	0.175301	0.057240	0.029*	
N1	0.02611 (9)	0.4958 (4)	0.30532 (8)	0.0226 (5)	
N2	0.16422 (8)	0.3389 (4)	0.18865 (7)	0.0207 (5)	
O1	0.22911 (7)	−0.0059 (4)	0.20281 (6)	0.0246 (4)	
O2	0.02475 (7)	0.6650 (3)	0.27241 (6)	0.0258 (4)	
O3	−0.01168 (8)	0.4766 (4)	0.33673 (7)	0.0300 (4)	
F1	0.18323 (6)	−0.4115 (3)	0.37631 (5)	0.0285 (4)	
F2	0.17958 (7)	−0.0767 (3)	0.42334 (5)	0.0310 (4)	
F3	0.09820 (6)	−0.2979 (3)	0.40528 (5)	0.0289 (4)	
S1	0.10703 (2)	0.51050 (12)	0.21373 (2)	0.02003 (18)	

### Atomic displacement parameters (Å^2^)

**Table:**

	*U*^11^	*U*^22^	*U*^33^	*U*^12^	*U*^13^	*U*^23^
C3	0.0068 (10)	0.0277 (13)	0.0242 (13)	−0.0001 (10)	−0.0013 (9)	−0.0037 (10)
C3A	0.0076 (10)	0.0246 (13)	0.0222 (12)	−0.0019 (9)	−0.0016 (9)	−0.0043 (10)
C4	0.0064 (10)	0.0272 (14)	0.0252 (12)	−0.0005 (9)	−0.0017 (9)	−0.0032 (10)
C5	0.0092 (11)	0.0281 (14)	0.0216 (12)	−0.0024 (9)	−0.0023 (9)	−0.0037 (10)
C6	0.0071 (10)	0.0309 (14)	0.0225 (12)	−0.0028 (10)	0.0006 (9)	−0.0050 (10)
C7	0.0069 (10)	0.0263 (13)	0.0254 (12)	−0.0007 (9)	−0.0005 (9)	−0.0052 (10)
C7A	0.0079 (10)	0.0250 (13)	0.0223 (12)	−0.0004 (9)	−0.0043 (9)	−0.0048 (10)
C8	0.0114 (11)	0.0308 (14)	0.0231 (12)	−0.0011 (10)	0.0029 (9)	−0.0044 (11)
C9	0.0098 (11)	0.0313 (14)	0.0211 (12)	−0.0002 (10)	0.0018 (9)	0.0016 (10)
C10	0.0089 (10)	0.0295 (14)	0.0240 (12)	−0.0012 (10)	−0.0011 (9)	0.0017 (10)
C11	0.0081 (11)	0.0362 (14)	0.0258 (13)	0.0000 (10)	0.0005 (9)	0.0032 (11)
C12	0.0101 (11)	0.0443 (17)	0.0287 (13)	−0.0033 (11)	−0.0020 (10)	0.0013 (12)
C13	0.0143 (12)	0.0441 (17)	0.0259 (13)	−0.0040 (11)	−0.0035 (10)	−0.0006 (12)
C14	0.0178 (12)	0.0401 (16)	0.0253 (13)	0.0008 (11)	0.0008 (10)	−0.0029 (12)
C15	0.0112 (11)	0.0374 (15)	0.0249 (13)	0.0014 (10)	0.0009 (9)	−0.0005 (11)
N1	0.0092 (10)	0.0306 (12)	0.0275 (11)	0.0023 (8)	−0.0015 (8)	−0.0055 (10)
N2	0.0100 (9)	0.0278 (11)	0.0243 (11)	0.0004 (8)	0.0002 (8)	0.0002 (9)
O1	0.0121 (8)	0.0344 (10)	0.0277 (9)	0.0061 (7)	0.0051 (7)	0.0018 (8)
O2	0.0152 (8)	0.0298 (10)	0.0319 (10)	0.0052 (7)	−0.0024 (7)	−0.0004 (8)
O3	0.0132 (9)	0.0445 (12)	0.0331 (10)	0.0060 (8)	0.0074 (8)	−0.0038 (9)
F1	0.0221 (7)	0.0346 (9)	0.0291 (8)	0.0088 (6)	0.0047 (6)	0.0042 (7)
F2	0.0263 (8)	0.0426 (9)	0.0229 (7)	−0.0050 (7)	−0.0073 (6)	−0.0018 (7)
F3	0.0131 (7)	0.0415 (9)	0.0326 (8)	−0.0026 (6)	0.0060 (6)	0.0070 (7)
S1	0.0096 (3)	0.0265 (3)	0.0238 (3)	0.0017 (2)	−0.0003 (2)	−0.0008 (3)

### Geometric parameters (Å, º)

**Table:**

C3—O1	1.226 (3)	C10—C15	1.526 (3)
C3—N2	1.366 (3)	C10—C11	1.533 (3)
C3—C3A	1.463 (3)	C10—H10	1.0000
C3A—C4	1.384 (3)	C11—C12	1.521 (3)
C3A—C7A	1.402 (3)	C11—H11A	0.9900
C4—C5	1.388 (3)	C11—H11B	0.9900
C4—H4	0.9500	C12—C13	1.519 (3)
C5—C6	1.395 (3)	C12—H12A	0.9900
C5—C8	1.488 (3)	C12—H12B	0.9900
C6—C7	1.374 (3)	C13—C14	1.523 (3)
C6—H6	0.9500	C13—H13A	0.9900
C7—C7A	1.391 (3)	C13—H13B	0.9900
C7—N1	1.448 (3)	C14—C15	1.523 (3)
C7A—S1	1.706 (2)	C14—H14A	0.9900
C8—F1	1.333 (3)	C14—H14B	0.9900
C8—F2	1.343 (3)	C15—H15A	0.9900
C8—F3	1.345 (3)	C15—H15B	0.9900
C9—N2	1.464 (3)	N1—O3	1.228 (3)
C9—C10	1.528 (3)	N1—O2	1.241 (3)
C9—H9A	0.9900	N2—S1	1.709 (2)
C9—H9B	0.9900		
			
O1—C3—N2	123.8 (2)	C11—C10—H10	107.8
O1—C3—C3A	127.7 (2)	C12—C11—C10	111.2 (2)
N2—C3—C3A	108.44 (19)	C12—C11—H11A	109.4
C4—C3A—C7A	121.2 (2)	C10—C11—H11A	109.4
C4—C3A—C3	126.9 (2)	C12—C11—H11B	109.4
C7A—C3A—C3	112.0 (2)	C10—C11—H11B	109.4
C3A—C4—C5	119.0 (2)	H11A—C11—H11B	108.0
C3A—C4—H4	120.5	C13—C12—C11	111.3 (2)
C5—C4—H4	120.5	C13—C12—H12A	109.4
C4—C5—C6	121.0 (2)	C11—C12—H12A	109.4
C4—C5—C8	120.6 (2)	C13—C12—H12B	109.4
C6—C5—C8	118.4 (2)	C11—C12—H12B	109.4
C7—C6—C5	118.9 (2)	H12A—C12—H12B	108.0
C7—C6—H6	120.6	C12—C13—C14	110.9 (2)
C5—C6—H6	120.6	C12—C13—H13A	109.5
C6—C7—C7A	121.8 (2)	C14—C13—H13A	109.5
C6—C7—N1	120.5 (2)	C12—C13—H13B	109.5
C7A—C7—N1	117.6 (2)	C14—C13—H13B	109.5
C7—C7A—C3A	118.1 (2)	H13A—C13—H13B	108.0
C7—C7A—S1	129.07 (18)	C15—C14—C13	111.2 (2)
C3A—C7A—S1	112.84 (18)	C15—C14—H14A	109.4
F1—C8—F2	106.21 (18)	C13—C14—H14A	109.4
F1—C8—F3	106.9 (2)	C15—C14—H14B	109.4
F2—C8—F3	106.07 (18)	C13—C14—H14B	109.4
F1—C8—C5	113.21 (19)	H14A—C14—H14B	108.0
F2—C8—C5	112.1 (2)	C14—C15—C10	111.6 (2)
F3—C8—C5	111.90 (18)	C14—C15—H15A	109.3
N2—C9—C10	113.45 (19)	C10—C15—H15A	109.3
N2—C9—H9A	108.9	C14—C15—H15B	109.3
C10—C9—H9A	108.9	C10—C15—H15B	109.3
N2—C9—H9B	108.9	H15A—C15—H15B	108.0
C10—C9—H9B	108.9	O3—N1—O2	124.3 (2)
H9A—C9—H9B	107.7	O3—N1—C7	119.6 (2)
C15—C10—C9	109.99 (19)	O2—N1—C7	116.06 (19)
C15—C10—C11	110.53 (19)	C3—N2—C9	122.1 (2)
C9—C10—C11	112.6 (2)	C3—N2—S1	116.46 (16)
C15—C10—H10	107.8	C9—N2—S1	121.45 (16)
C9—C10—H10	107.8	C7A—S1—N2	90.24 (10)
			
O1—C3—C3A—C4	2.0 (4)	C6—C5—C8—F3	41.5 (3)
N2—C3—C3A—C4	−178.5 (2)	N2—C9—C10—C15	−161.7 (2)
O1—C3—C3A—C7A	−177.3 (2)	N2—C9—C10—C11	74.5 (3)
N2—C3—C3A—C7A	2.3 (3)	C15—C10—C11—C12	55.4 (3)
C7A—C3A—C4—C5	0.9 (3)	C9—C10—C11—C12	178.9 (2)
C3—C3A—C4—C5	−178.2 (2)	C10—C11—C12—C13	−56.3 (3)
C3A—C4—C5—C6	2.4 (3)	C11—C12—C13—C14	56.3 (3)
C3A—C4—C5—C8	−175.7 (2)	C12—C13—C14—C15	−55.7 (3)
C4—C5—C6—C7	−2.8 (3)	C13—C14—C15—C10	55.6 (3)
C8—C5—C6—C7	175.3 (2)	C9—C10—C15—C14	179.9 (2)
C5—C6—C7—C7A	0.0 (3)	C11—C10—C15—C14	−55.2 (3)
C5—C6—C7—N1	179.4 (2)	C6—C7—N1—O3	−9.8 (3)
C6—C7—C7A—C3A	3.2 (3)	C7A—C7—N1—O3	169.7 (2)
N1—C7—C7A—C3A	−176.3 (2)	C6—C7—N1—O2	170.6 (2)
C6—C7—C7A—S1	−178.69 (19)	C7A—C7—N1—O2	−9.9 (3)
N1—C7—C7A—S1	1.9 (3)	O1—C3—N2—C9	0.7 (3)
C4—C3A—C7A—C7	−3.6 (3)	C3A—C3—N2—C9	−178.87 (19)
C3—C3A—C7A—C7	175.6 (2)	O1—C3—N2—S1	178.73 (18)
C4—C3A—C7A—S1	177.93 (18)	C3A—C3—N2—S1	−0.8 (2)
C3—C3A—C7A—S1	−2.8 (2)	C10—C9—N2—C3	91.3 (3)
C4—C5—C8—F1	−19.4 (3)	C10—C9—N2—S1	−86.6 (2)
C6—C5—C8—F1	162.4 (2)	C7—C7A—S1—N2	−176.2 (2)
C4—C5—C8—F2	100.7 (3)	C3A—C7A—S1—N2	1.96 (17)
C6—C5—C8—F2	−77.5 (3)	C3—N2—S1—C7A	−0.63 (18)
C4—C5—C8—F3	−140.3 (2)	C9—N2—S1—C7A	177.45 (19)

### Hydrogen-bond geometry (Å, º)

**Table:**

*D*—H···*A*	*D*—H	H···*A*	*D*···*A*	*D*—H···*A*
C4—H4···O1^i^	0.95	2.25	3.193 (3)	175

Symmetry code: (i) −*x*+1/2, −*y*−1/2, −*z*+1/2.
