# Supplementary material for: Crystal structure and anti­mycobacterial evaluation of 2-(cyclo­hexyl­meth­yl)-7-nitro-5-(tri­fluoro­meth­yl)benzo[d]iso­thia­zol-3(2H)-one
Source: Acta Crystallogr E Crystallogr Commun. 2023 Nov 30;79(Pt 12):1194–8. doi: 10.1107/S2056989023010137 (PMC10833412; doi:10.1107/S2056989023010137)
Supplement: Supplementary file 4 [file e-79-01194-sup4.pdf]

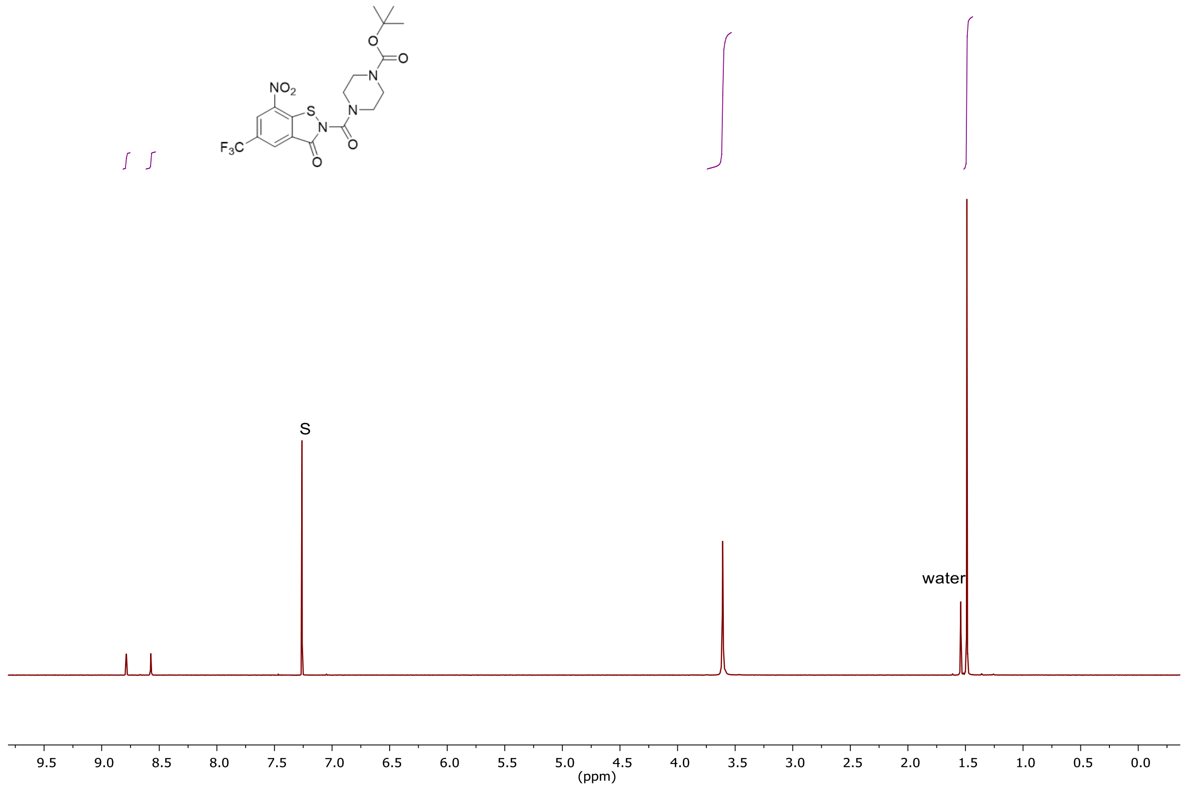

Figure S1 ^{1}H NMR spectrum of 2-(4-Boc-piperazine-1-carbonyl)-7-nitro-5-(trifluoromethyl)-benzo[*d*]isothiazol-3(2*H*)-one in chloroform-*d*. S denotes the residual solvent signal.

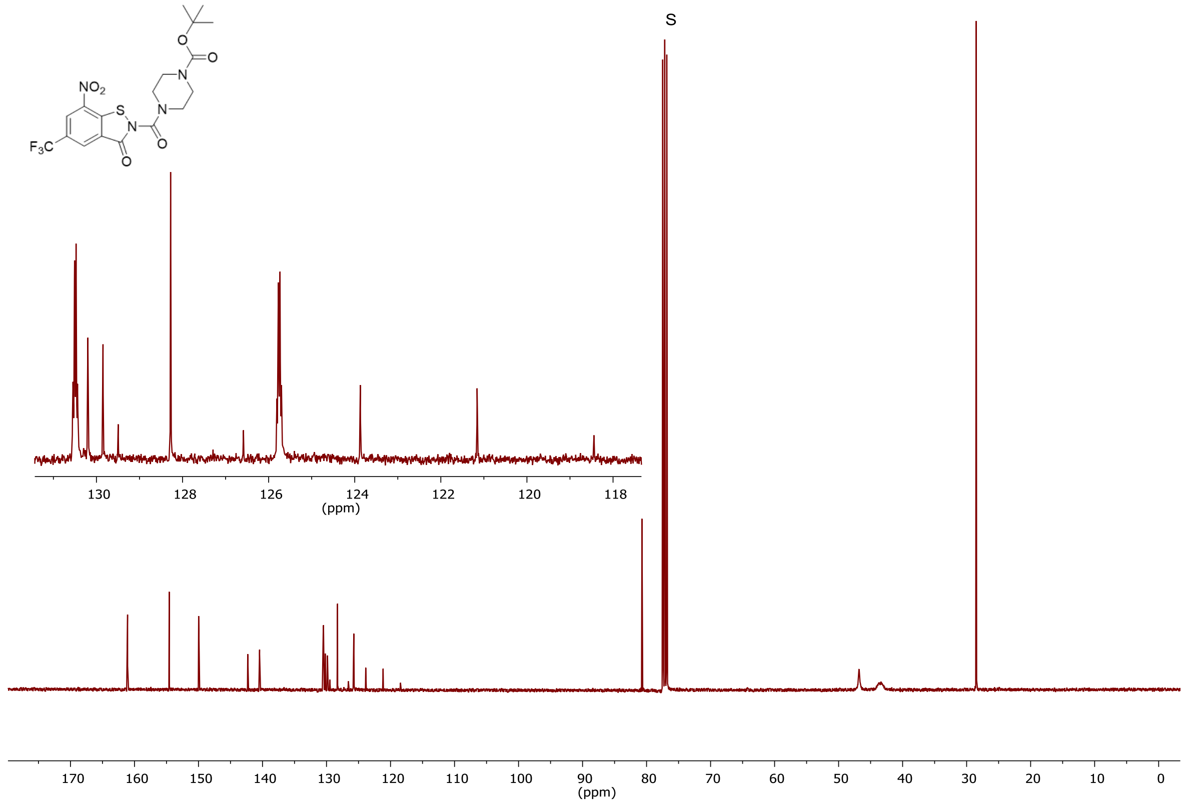

Figure S2 ^{13}C NMR spectrum of 2-(4-Boc-piperazine-1-carbonyl)-7-nitro-5-(trifluoromethyl)-benzo[*d*]isothiazol-3(2*H*)-one in chloroform-*d*. S denotes the residual solvent signal.

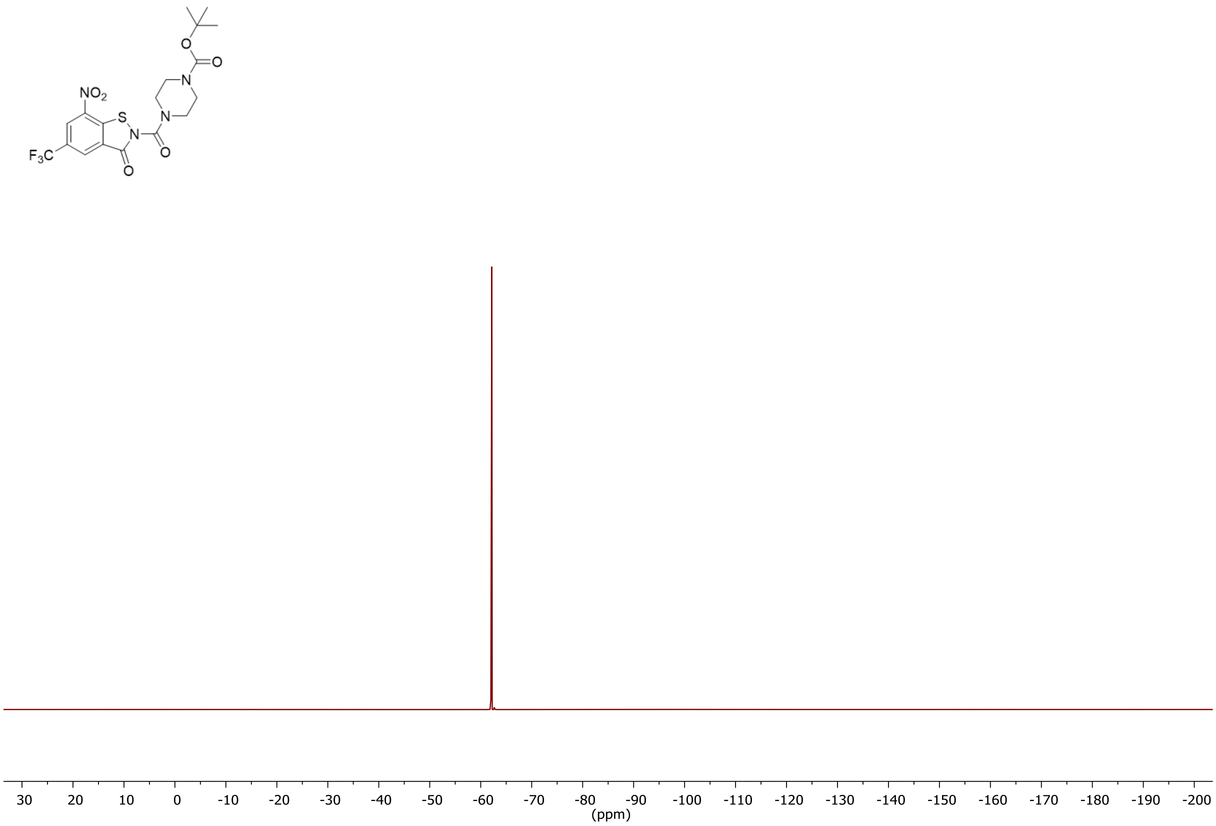

Figure S3 ^{19}F NMR spectrum of 2-(4-Boc-piperazine-1-carbonyl)-7-nitro-5-(trifluoromethyl)-benzo[*d*]isothiazol-3(2*H*)-one in chloroform-*d*.

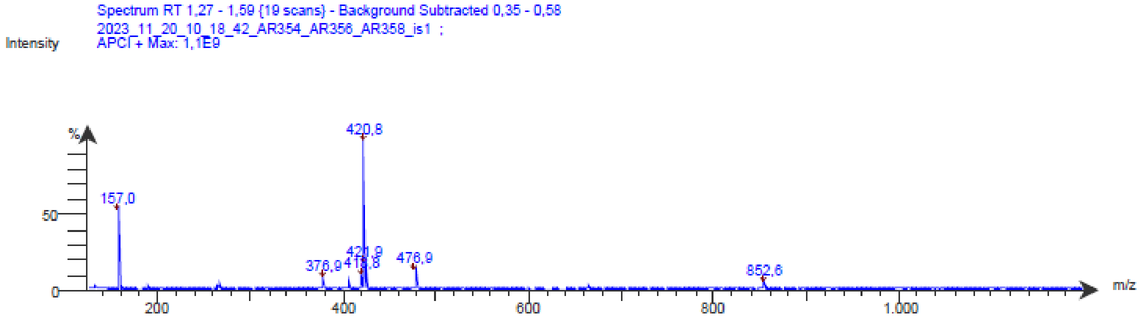

Figure S4 APCI^{+} mass spectrum of 2-(4-Boc-piperazine-1-carbonyl)-7-nitro-5-(trifluoro-methyl)benzo[*d*]isothiazol-3(2*H*)-one.

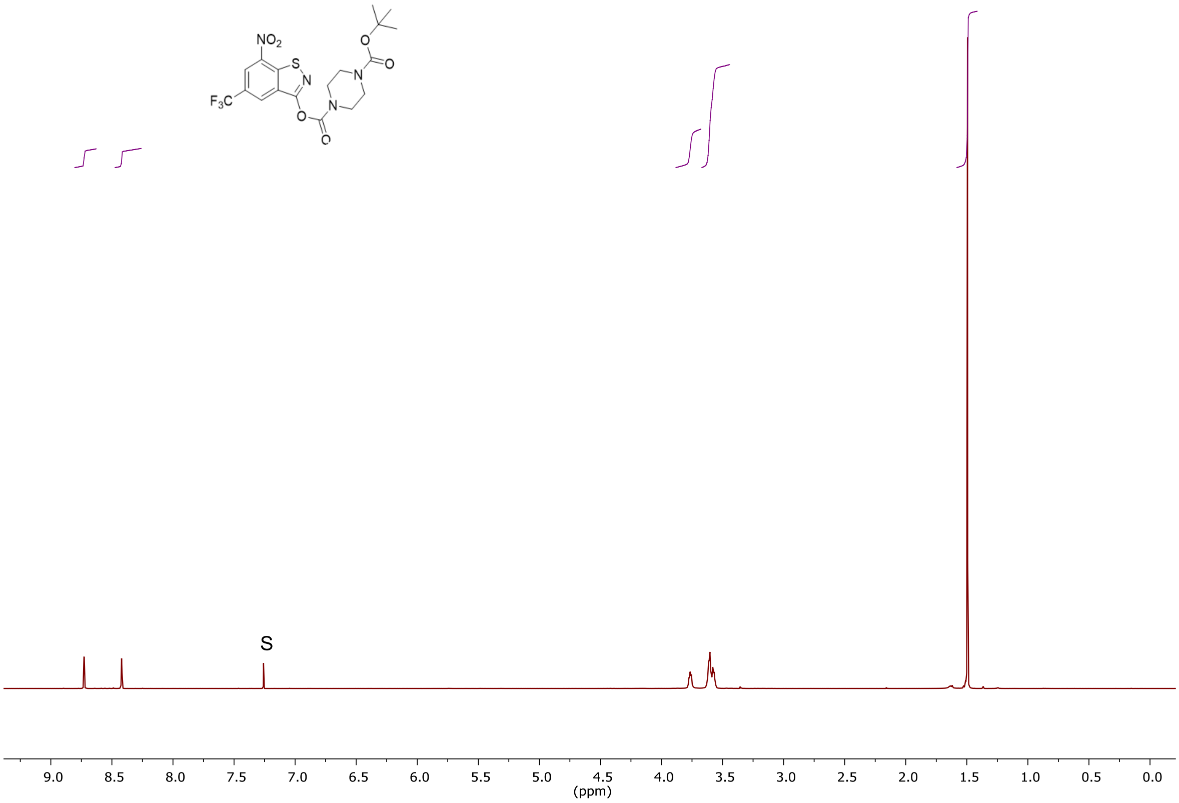

Figure S5 ^{1}H NMR spectrum of 7-nitro-5-(trifluoromethyl)benzo[*d*]isothiazol-3-yl 4-Boc-piperazine-1-carboxylate in chloroform-*d*. S denotes the residual solvent signal.

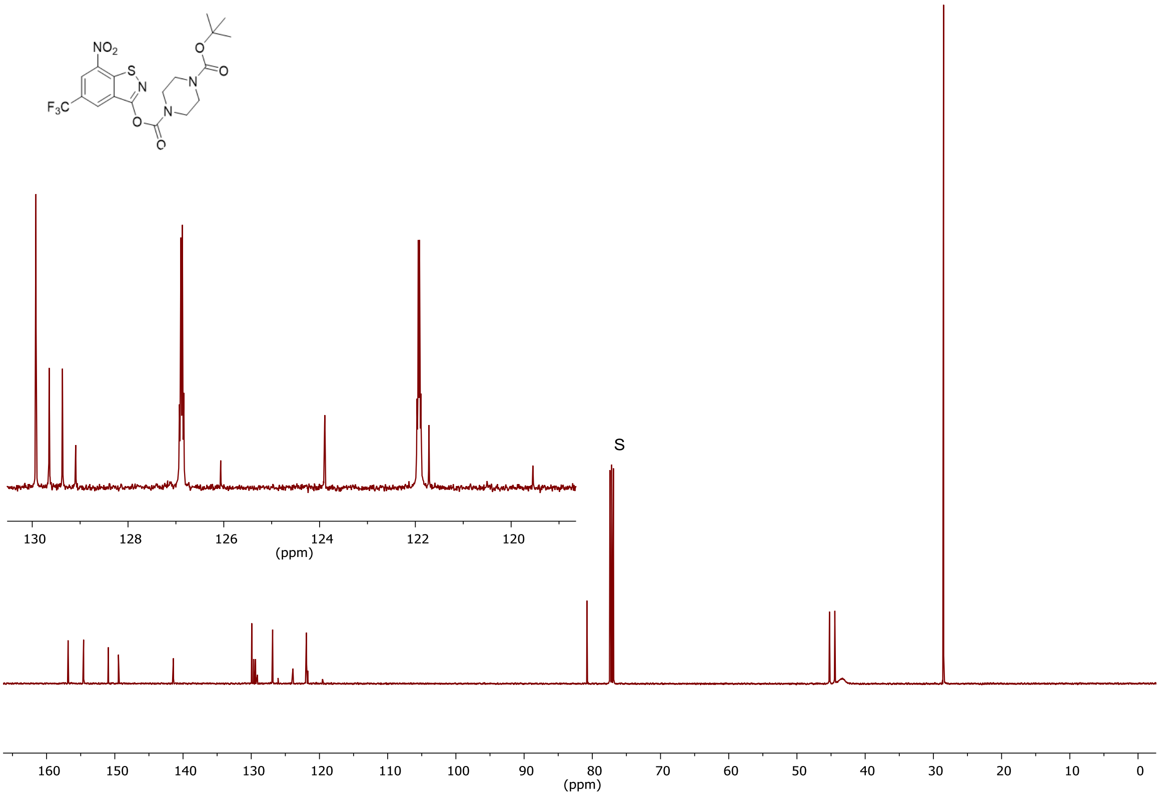

Figure S6 ^{13}C NMR spectrum of 7-nitro-5-(trifluoromethyl)benzo[*d*]isothiazol-3-yl 4-Boc-piperazine-1-carboxylate in chloroform-*d*. S denotes the residual solvent signal.

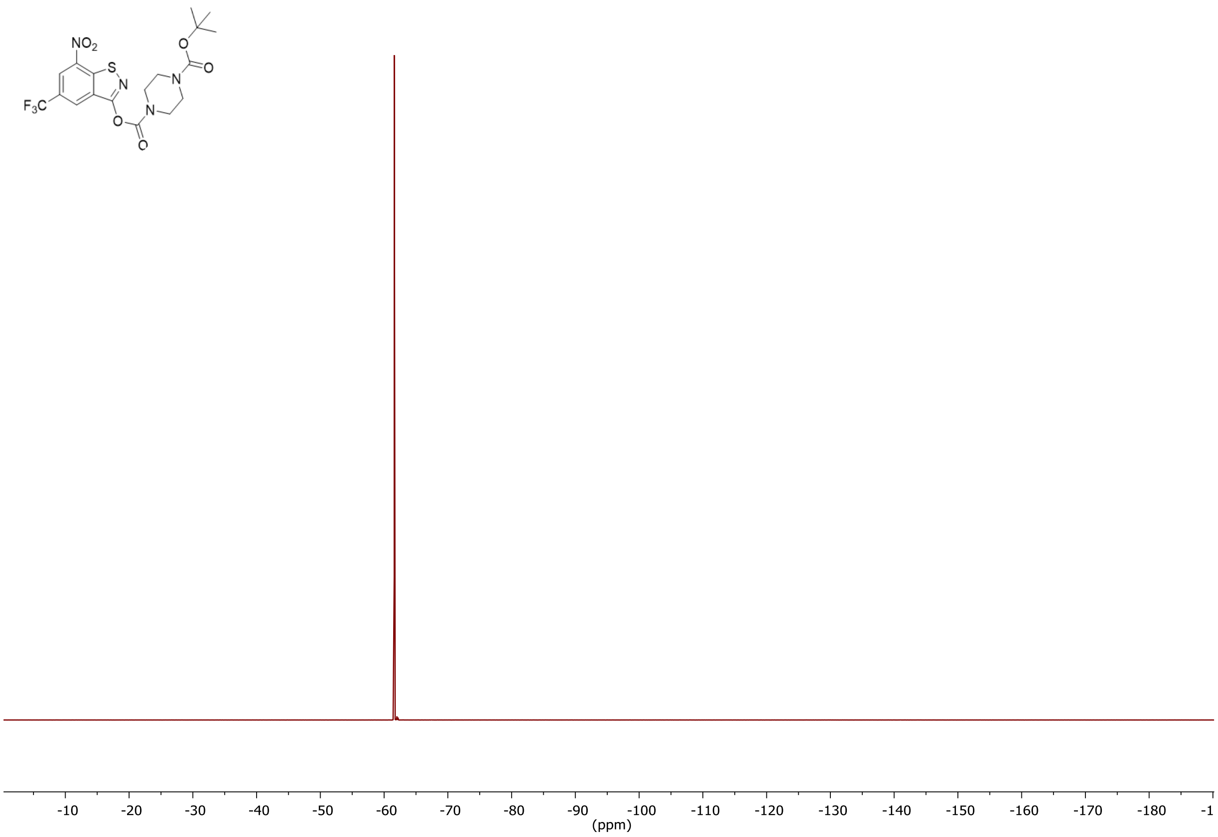

Figure S7 ^{19}F NMR spectrum of 7-nitro-5-(trifluoromethyl)benzo[*d*]isothiazol-3-yl 4-Boc-piperazine-1-carboxylate in chloroform-*d*.

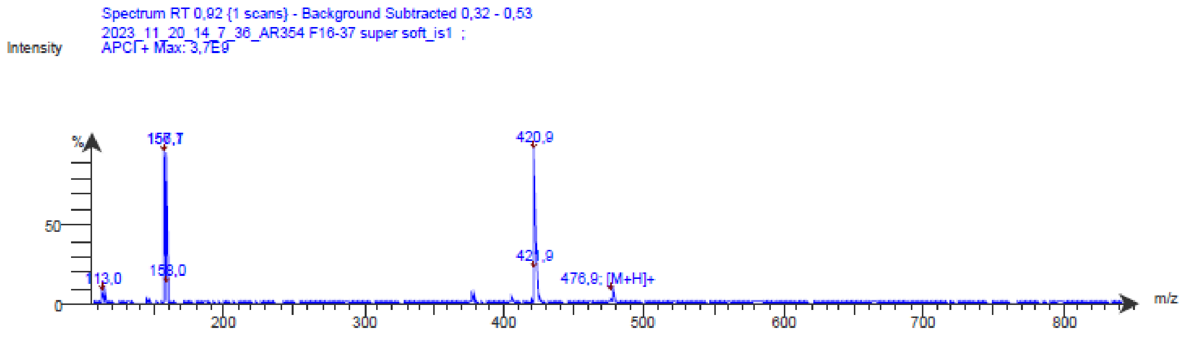

Figure S8 APCI$^+$ mass spectrum of 7-nitro-5-(trifluoromethyl)benzo[*d*]isothiazol-3-yl 4-Boc-piperazine-1-carboxylate.

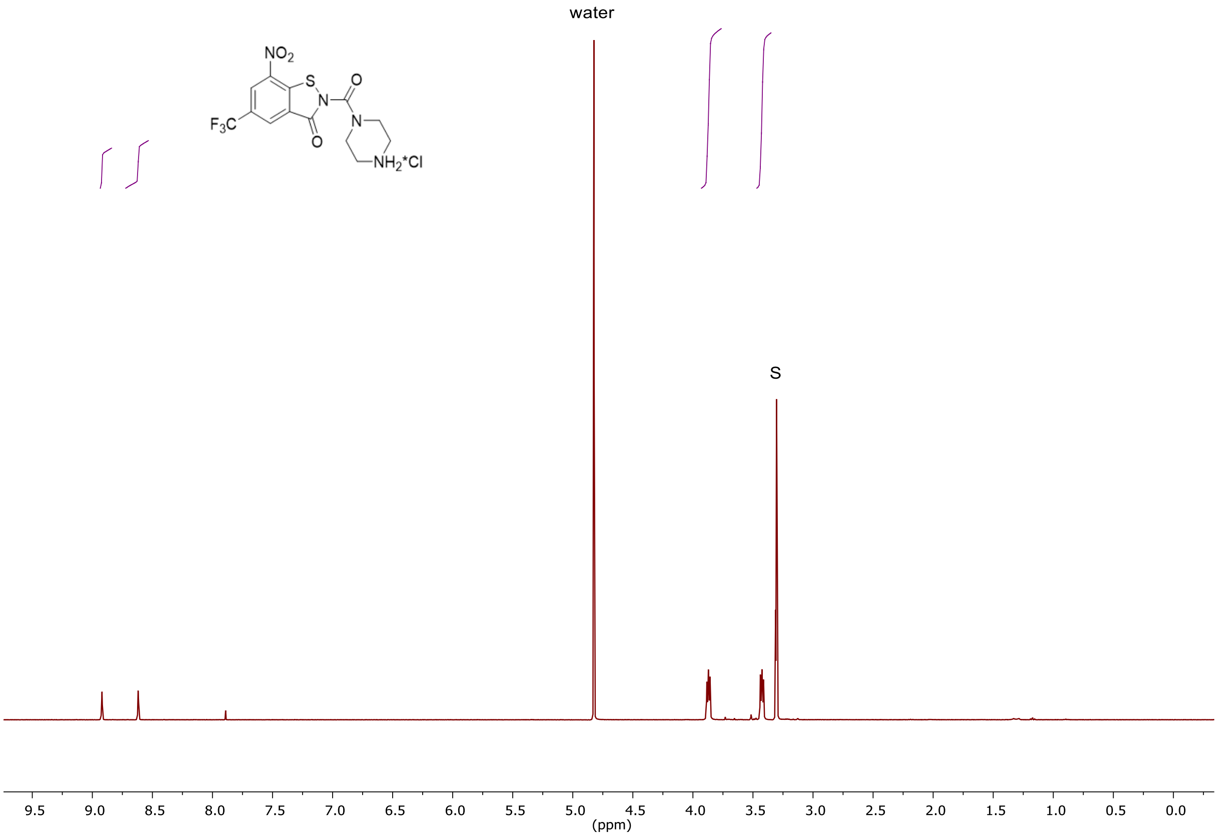

Figure S9 ^{1}H NMR spectrum of 4-(7-nitro-3-oxo-5-(trifluoromethyl)-2,3-dihydrobenzo[*d*]-isothiazole-2-carbonyl)piperazin-1-ium chloride (**2**) in methanol-d_4. S denotes the residual solvent signal.

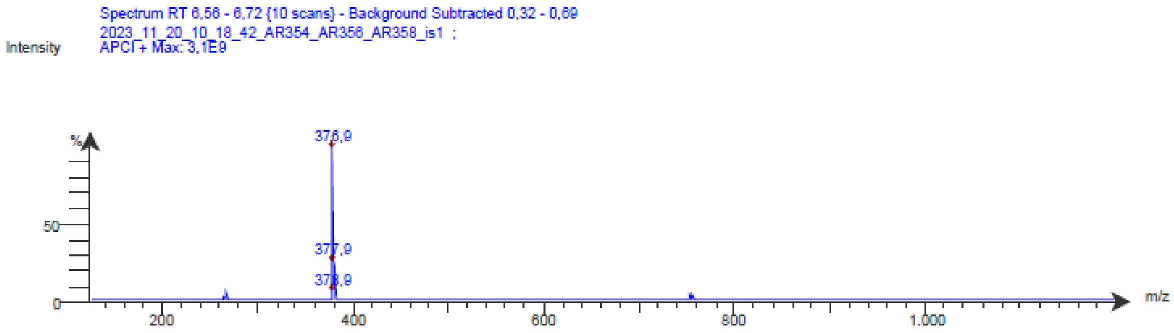

Figure S10 APCI^{+} mass spectrum of 4-(7-nitro-3-oxo-5-(trifluoromethyl)-2,3-dihydro-benzo[*d*]-isothiazole-2-carbonyl)piperazin-1-ium chloride (**2**).

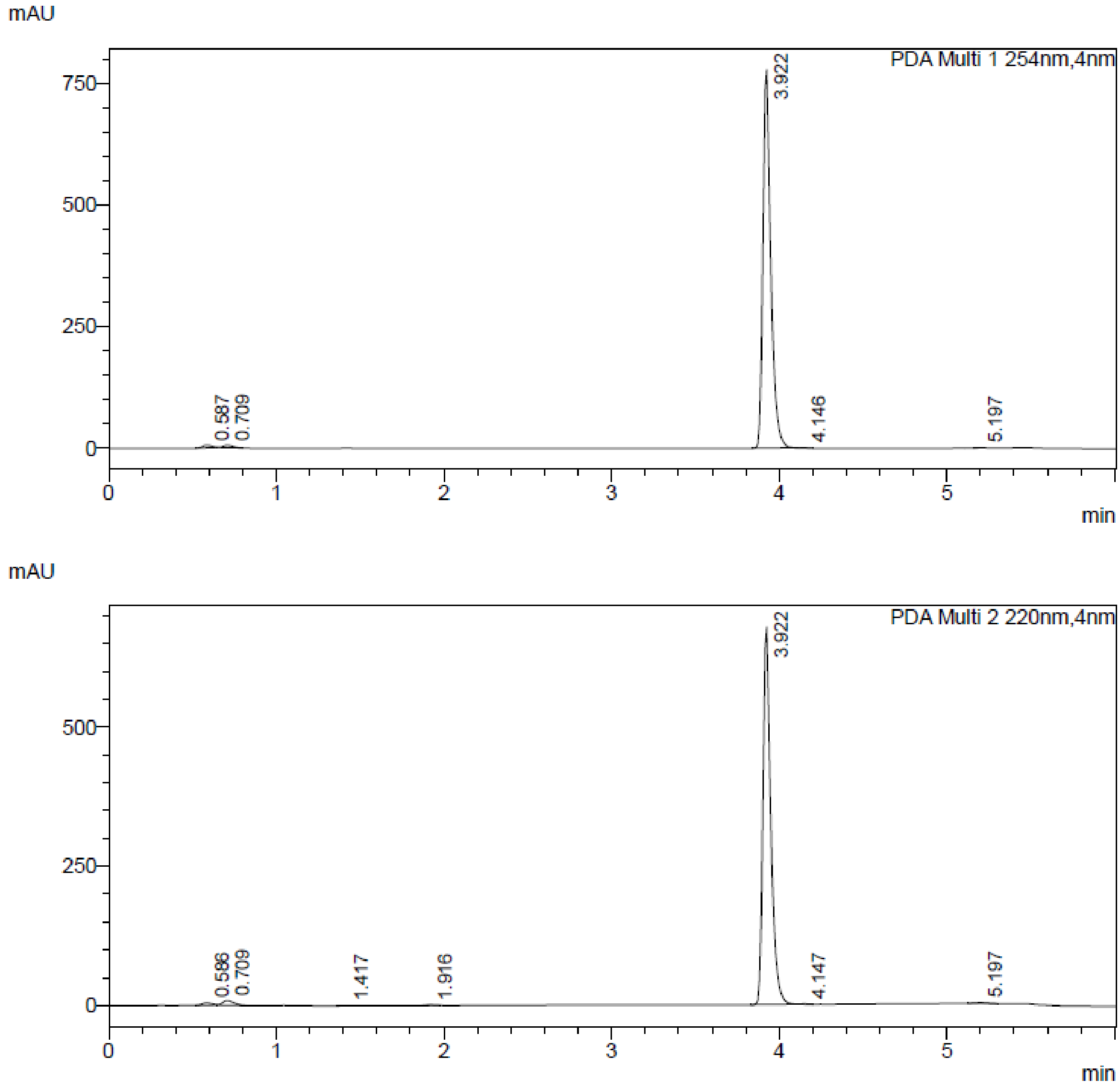

Figure S11 HPLC traces of 2-(cyclohexylmethyl)-7-nitro-5-(trifluoromethyl)benzo[*d*]-isothiazol-3(2*H*)-one (**4**) at 254 nm (top) and 220 nm (bottom).

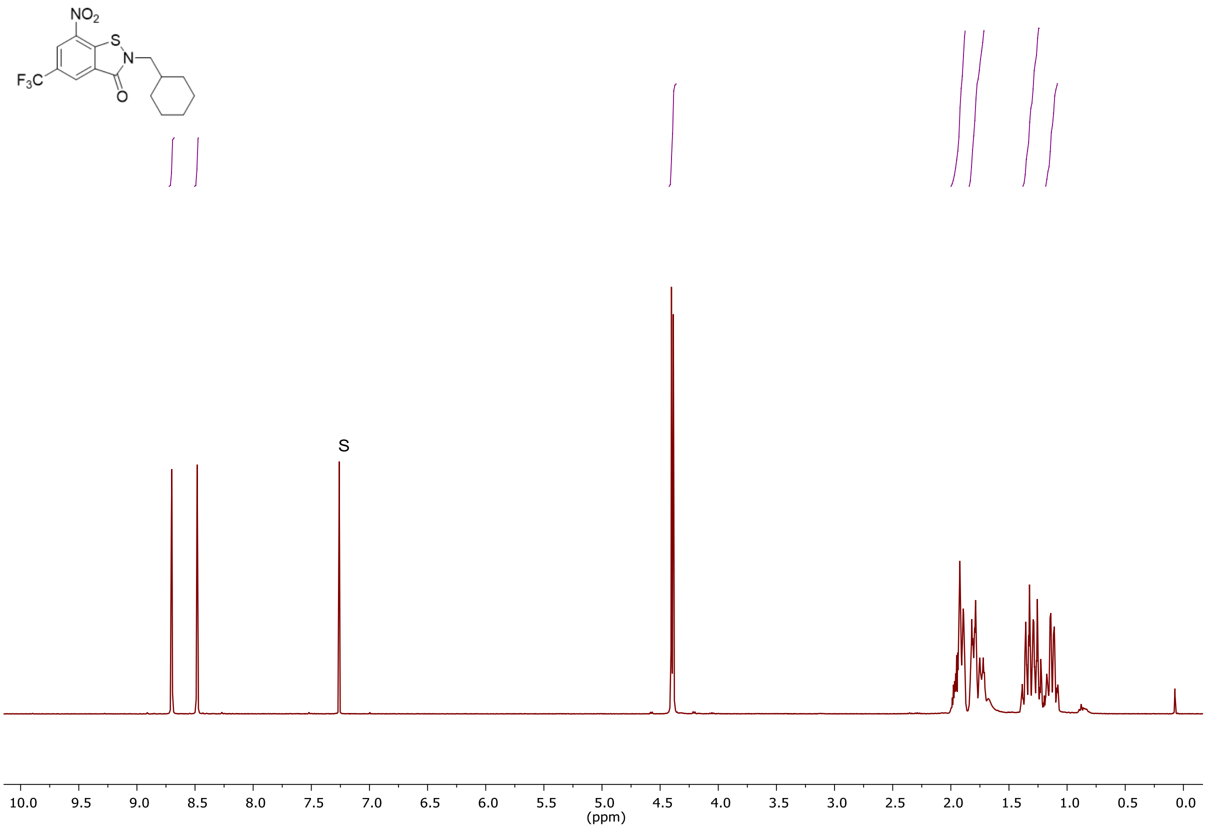

Figure S12 ^{1}H NMR spectrum of 2-(cyclohexylmethyl)-7-nitro-5-(trifluoromethyl)benzo[*d*]-isothiazol-3(2*H*)-one (**4**) in chloroform-*d*. S denotes the residual solvent signal.

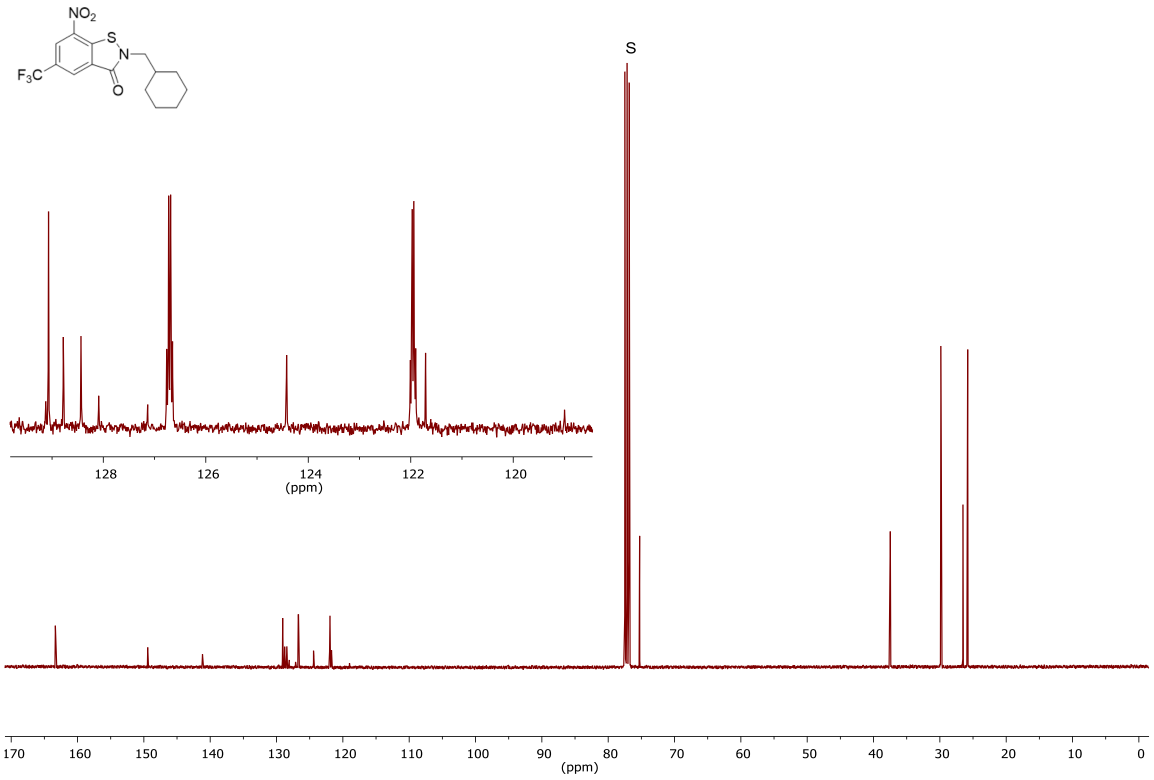

Figure S13 ^{13}C NMR spectrum of 2-(cyclohexylmethyl)-7-nitro-5-(trifluoromethyl)benzo[*d*]-isothiazol-3(2*H*)-one (**4**) in chloroform-*d*. S denotes the residual solvent signal.

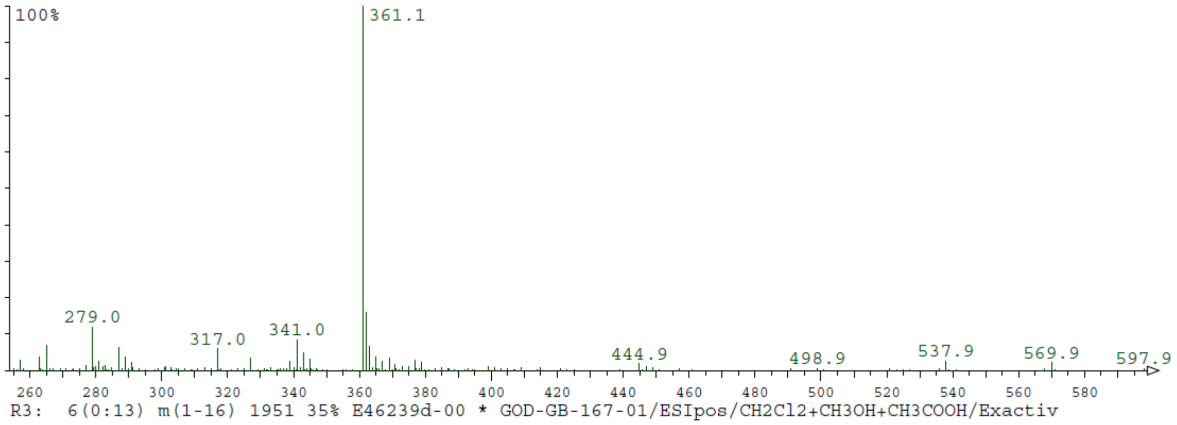


```
Mass to be matched (m/z): 361.08264  charge: 1

Mass tolerance: ±0.002
restriction of atom numbers:
      C        H        N       O       F       S
   1-100    1-100     0-2    0-10     0-3     1-1
Number of calculated formulas: 4
Formula                Diff. (ppm)  theor. m/z
C14 H19 N1 O8 S1            -0.14   361.08259
C15 H16 F3 N2 O3 S1          0.50   361.08282  <-
C18 H15 F2 N2 O2 S1         -2.66   361.08168
C11 H20 F1 N1 O9 S1          3.02   361.08373
```

Figure S14 High resolution ESI$^+$ mass spectrum of 2-(cyclohexylmethyl)-7-nitro-5-(trifluoromethyl)benzo[*d*]-isothiazol-3(2*H*)-one (**4**) in dichloromethane/methanol/acetic acid.

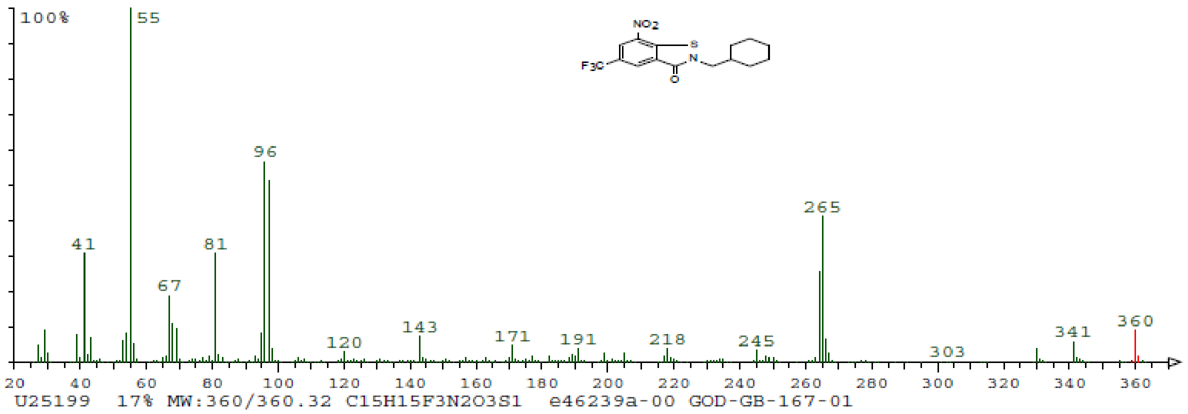

Figure S15 EI mass spectrum of 2-(cyclohexylmethyl)-7-nitro-5-(trifluoromethyl)benzo[*d*]-isothiazol-3(2*H*)-one (**4**).

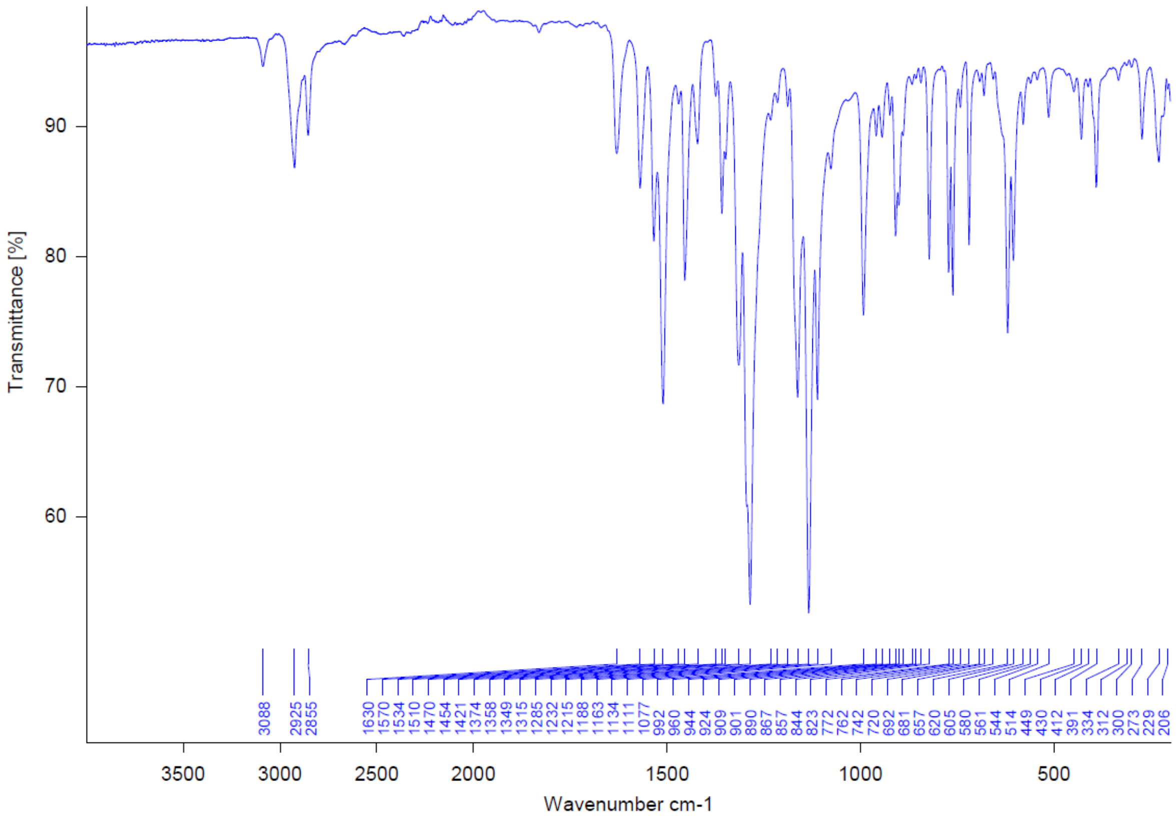

Figure S16 ATR-FT-IR spectrum of 2-(cyclohexylmethyl)-7-nitro-5-(trifluoromethyl)-benzo[*d*]-isothiazol-3(2*H*)-one (**4**).
